# A Polarization Independent Quasi-TEM Metamaterial Absorber for X and Ku Band Sensing Applications

**DOI:** 10.3390/s18124209

**Published:** 2018-11-30

**Authors:** Ahasanul Hoque, Mohammad Tariqul Islam, Ali F. Almutairi, Touhidul Alam, Mandeep Jit Singh, Nowshad Amin

**Affiliations:** 1 Centre of Advanced Electronic and Communication Engineering, Faculty of Engineering and Built Environment, Universiti Kebangsaan Malaysia, Bangi 43600, Selangor, Malaysia; touhid13@siswa.ukm.edu.my (T.A.); mandeep@ukm.edu.my (M.J.S.); 2 Electrical Engineering Department, Kuwait University, Kuwait City 13060, Kuwait; 3 Institute of Sustainable Energy (ISE), Universiti Tenaga Nasional, Jalan IKRAM-UNITEN, Kajang 43000, Selangor, Malaysia; nowshad@uniten.edu.my

**Keywords:** absorber, dual-band, metamaterial, quasi-TEM, sensing, X band

## Abstract

In this paper, a dual-band metamaterial absorber (MMA) ring with a mirror reflexed C-shape is introduced for X and Ku band sensing applications. The proposed metamaterial consists of two square ring resonators and a mirror reflexed C-shape, which reveals two distinctive absorption bands in the electromagnetic wave spectrum. The mechanism of the two-band absorber particularly demonstrates two resonance frequencies and absorption was analyzed using a quasi-TEM field distribution. The absorption can be tunable by changing the size of the metallic ring in the frequency spectrum. Design and analysis of the proposed meta-absorber was performed using the finite-integration technique (FIT)-based CST microwave studio simulation software. Two specific absorption peaks value of 99.6% and 99.14% are achieved at 13.78 GHz and 15.3 GHz, respectively. The absorption results have been measured and compared with computational results. The proposed dual-band absorber has potential applications in sensing techniques for satellite communication and radar systems.

## 1. Introduction

Periodic arrangement of metallic patterns in the microwave frequency range using thin wires and certain design characteristics show unconventional properties of materials like negative values of permittivity, permeability, refractive index, etc. These characteristics-based materials are known as metamaterials and cannot be found in Nature. A significant number of researchers have been comprehensively studying these phenomena during the last few years observing such characteristics of metamaterials. Due to a wide range of application scope from cloaking to radar systems [1,2,3,4] the research interest of scholars is increasing day by day. For instance, a detailed review on photonic, terahertz and microwave applications was provided in [5], where the authors cited numerous mechanisms as well as materials regarding metamaterial and metadevice research. Robust analysis and sophisticated design for sensing based on the metamaterial concept was described in [6,7,8], such as nanoparticles-inspired structural properties analysis in terms of Maxwell’s equation and their application in sensing, structures with near-zero parameters (like permittivity, permeability or refractive index) working principle with application potentiality, the substantial role of grapheme in the inhomogeneous spatiality, and non-uniform conductivity pattern in transformation optics, etc. Remote sensing techniques using metamaterial absorbers is one of the promising research fields in recent years. Conventional remote sensors have performance issues in certain critical application environments whereas an absorber with metamaterial properties can overcome those issues [1].

Typically, a perfect metamaterial absorber consists of three different structures like two metallic layers namely one ground plane and an electric split ring resonator (SRR), where in between these there exists a dielectric layer. The resonator is intended to couple a strong uniform electric field with dielectric layer but a weak magnetic field is observed [2,3,4]. In this way, the electric field becomes dependent on the frequency range. Regarding the incident waves, the magnetic field propagates between the resonator and the metallic ground plane and incurs a frequency-dependent magnetic response. To get absorption properly, various measures can be taken like dimension variation of the resonator and ground plane or changes to the gap. As a result, a matching of impedance between the absorber and free space happens at that instant and simultaneously the reflection coefficient tries to reach almost zero. Typical absorbers are physically thick in size and their frequency response is very much hampered by their complex permittivity and permeability [2,3,4]. However, MM absorbers are frequently used in terahertz (THz), visible frequency spectrum, infrared range applications, etc. [9,10,11,12].

Common applications of MM absorbers in radar technology are to reduce the radar signature in stealth techniques and to overcome electromagnetic interference (EMI). This application requires a lowest possible thickness of material with a wider bandwidth for flexible operation and system integrity [13]. In the widespread application prospects, thickness and bandwidth are crucial parameters [14,15,16]. Other critical parameters are the magnetic loss spectrum and bandwidth optimization to minimize reflections. Structure-based analysis for making absorbers focused on cost optimization and light weight have been presented for microwave broadband electromagnetic absorption [17,18]. Other researchers have focused on the design of MM absorbers in the microwave scale. Such scholars designed and fabricated their unit cells in a millimeter range where some of them used SRR with a lumped resistor and got above 90% absorption [19,20,21]. Double negative (DNG) metamaterial-based, different permeability, and permittivity-based absorbers were also proposed by a few researchers. Those articles discussed the multiband operation, dense size, the various shape of the resonator with an irregular thickness of resistive sheets where absorption ranges from 50% to 90% approximately [22,23,24].

The recent trend to apply metamaterial absorbers in sensing applications represents a breakthrough in developing unique sensors for different engineering fields like biomedical applications, optics, chemical flow measurement, etc. Tunable metamaterial absorbers (TMAs) using a varactor diode were proposed for sensing applications where the dielectric constant is inversely proportional to the resonance frequency [25]. Ω-shaped multimode resonators presented in [26] have six-bands with an average absorption rate over 97.21%, but no sensing applications yet. Numerical and experimental demonstrations were performed using metamaterial absorbers where the authors [27,28] stated the sensing capability of material permittivity and pressure. A circular SRR-based metamaterial sensor was developed for microfluid, rotation and strain sensing but no application of absorption was mentioned [29]. Another researcher developed a microstrip metamaterial for liquid permittivity sensing [30] using a rectangular complementary-ring resonator (RCRR) but the Q factor of this unit is low. Single resonance is exhibited in a substrate-integrated waveguide (SIW) resonator where resonance frequency controlled dielectric loading and hence it cannot be independently tunable [31].

Herein, we propose a metamaterial absorber for pressure sensing applications in the X and Ku-band. The metamaterial absorber has been fabricated on a FR-4 (lossy) substrate (thickness *t* = 1.575 mm) which is a glass-reinforced NEMA-grade epoxy laminated material. These bands are used for satellite communications, especially tracking data relay satellite used for both the Space Shuttle and the International Space Station (ISS). Splitting the band into multiple segments varying by geographical region can be used for radio astronomy services, space research services, mobile satellite services, etc. The relative permittivity and permeability are −0.4213 and −1.251, respectively, at the highest absorption peak which is 99.6% (at frequency 13.78 GHz). This absorber was designed and simulated through the commercially available CST Microwave studio 2017 software. Details of the design procedure, and simulation steps are available in [32]. The design was intended to create a left-handed metamaterial (LHM) in X and Ku-band with maximum absorption and, hence, the reflection coefficient is also negative.

## 2. Design Methodology

An analytical model of a metamaterial absorber is a good method to describe it for better understanding of its basic design. There are different analytical models studied so far by researchers around the world. Some of them are multilayer and some are single layer. For instance, multilayer wire-based 2D thin material terminations [33] is one of the analytical method where authors characterized the design by complex surface conductivity. Full-wave numerical simulations verified this model. Metal nanoparticles have exotic optical properties and a new layered nanoparticle analytical model studied in [34] is a spherical structure for sensing applications. Another studied model is for infrared and optical regimes where the absorption was studied using both analytical and numerical methods. Here authors have used Transmission Line Theory and the Epsilon-near-zero (ENZ) property of the material to perform as an absorber [35]. This paper followed the same Transmission Line Theory to get a simplified analytical model in the GHz range. An advantage of this method is that it is a very straightforward and conventional circuit theory to develop the analysis.

**Figure 1:**
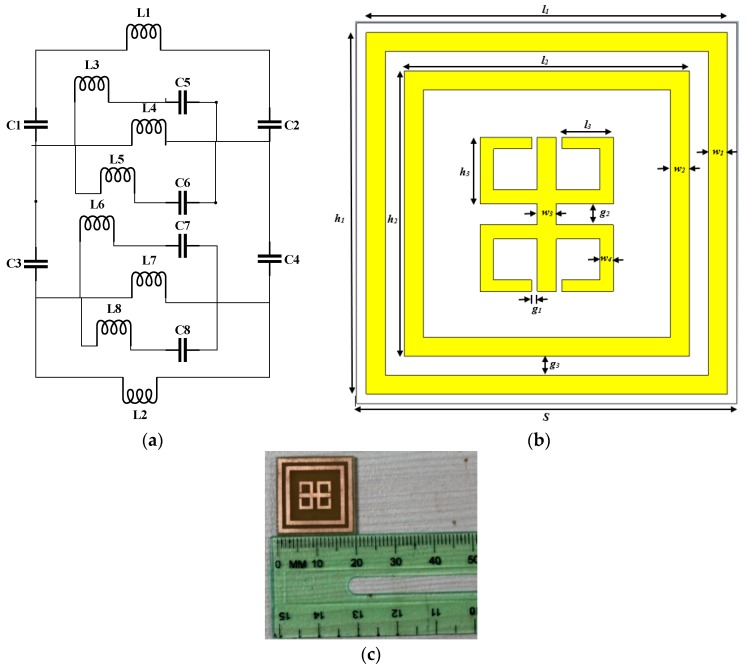
Proposed metamaterial absorber design (**a**) Equivalent circuit of prototype (**b**) unit cell absorber with dimension, and (**c**) fabricated prototype.

The proposed MM absorber consists of two ring-shaped resonators and inside those two-mirror reflexed C shape resonators are embossed on the dielectric material with a solid ground plane behind the substrate. A front view in structural representation with measurement details of the proposed absorber is depicted in Figure 1a–c. The metallic elements of the absorber are made of copper with thickness 0.035 mm, substrate thickness, *t* = 1.575 mm and this suggested absorber is developed on a single FR-4 printed circuit board (PCB) layer. FR-4 is a cost-effective fabrication material for application purposes. Simulation and measurement have demonstrated that the judgment, versatility and scope of work of the proposed design can be enhanced. Optimized geometrical dimensions of the proposed unit cell MM absorber is presented in Table 1.

For microstrip MM absorber design, a transmission line model was first analyzed for a prototype shown in Figure 1a,b that is a unit cell absorber with specific dimensions. The fabricated unit cell absorber is shown in Figure 1c. Following a typical approach for microstrip circuit, inductor and capacitor specifically depend on lumped components were referred to for modeling the design. Additionally, lumped components have significant characteristics regarding their values and dependency, as they are closely related to the Q factor and resonance frequency. Moreover, as the prototype contains a ground plane, the inductance decreases as it gets closer to the ground plane [36]. In Figure 1a inductor L1 to L8 and capacitor C1 to C8 represent the equivalent microstrip circuit model. To calculate the equivalent or total inductance and capacitance, transmission line principle mentioned in [37] gives the formula:

(1) $$L(\mathrm{nH})=2\times 10^{-4}l[\ln(\frac{l}{w+t})+1.193+0.02235\frac{w+t}{l}]K_{g}$$

(2) $$C(\mathrm{pF})=\frac{10^{-3}\varepsilon_{rd}wl}{36\pi d}$$

where, *l* = length of microstrip line
*w* = width of microstrip line
*t* = thickness of microstrip line
correction factor = $K_{g}=0.57-0.145\ln\frac{w^{\prime }}{h^{\prime }}$
$w^{\prime }$ = substrate width
$h^{\prime }$ = substrate thickness
$\varepsilon_{rd}$ = dielectric constant of dielectric film
*d* = distance between mutual overlapping stripline

Width and thickness of the substrate taken as *w*′ and *h*′, respectively, to calculate a correction factor. Total inductance and capacitance based on the above equivalent circuital model gives *L* = $1.166\times 10^{-4}nH$ and *C* = 0.68 pF, respectively. As mentioned in Section 1, absorbers basically have three different layers, the ground plane of the absorber comprises copper (Cu) with thickness of 0.035 mm which gives zero transmission of incident wave during propagation. Hence, the absorbance calculations have been done as follows [13]:

(3) $$A=1-R=1-{\left|\frac{Y_{0}-Y_{in}}{Y_{0}+Y_{in}}\right|}^{2}$$

where *Y*_0_ and *Y_in_* = *Y*_1_ + *Y_A_* are the characteristic admittance of air and the input admittance of the MM absorber. The input admittances *Y*_1_, *Y_A_* are formulated as:

(4) $$Y_{1}=-jY_{d}\cot(kh)=-j\sqrt{\frac{\varepsilon_{r}\varepsilon_{0}}{\mu_{r}\mu_{0}}}\cot(kh)$$

(5) $$Y_{A}=\frac{1}{R+j\omega L-\frac{j}{\omega C}}$$

where, *Y_d_*, $\epsilon_{r}$, $\mu_{r}$ and *k* = *k*_0_$\surd (\epsilon_{r}\times \mu_{r}$) are the characteristics admittance, relative permittivity, relative permeability, and wave number of dielectric substrates, and $\epsilon_{0}$, $\mu_{0}$, and *k*_0_ are the permittivity, permeability, and wave number of free spaces, respectively.

It is clearly in Equation (3) that the transmittance parameter ${\left|S{\left(\omega \right)}_{21}\right|}^{2}$ or *T* at angular frequency *ω* is zero due to the complete copper ground plane. Most researchers did not put any second resonator inside the lattice, which suffers from a poor form factor. In the proposed absorber, this mirror reflexed C-shaped resonators form another set of resonances with effective absorbance parallel to the first one. This uniqueness is significant of the proposed design with increased form factor as for X and Ku-band satellites. Based on Equation (1), theoretically the transmittance should be zero, and in simulation most of the power is lost between the resonator gaps as the electric fields converge to each other. Details of this will be presented as experimental data in Section 3. However, in this article a finite-integration technique (FIT)-based simulation method was used for structural analysis of the absorber. At the initial stage, the polarization of the unit cell was a quasi-TEM mode where X-axis comprises electric field whereas magnetic field in the Y-axis and Z-axis propagation of the EM field. Tetrahedral mesh is being used for the frequency domain solver on the unit cell absorber.

**Figure 2:**
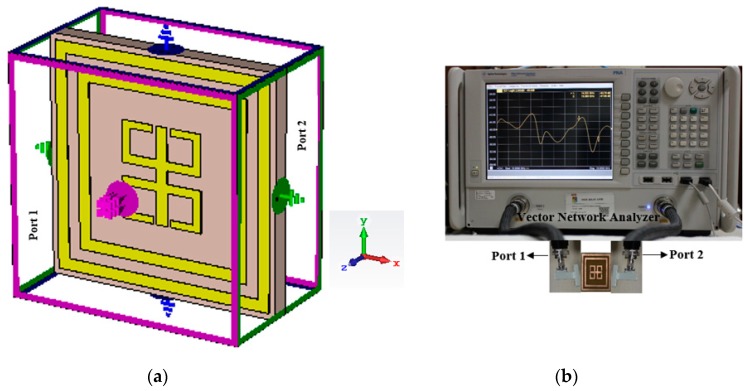
Prototype analysis (**a**) simulated boundary condition, (**b**) Experimental arrangement for measurement.

To verify the theoretical concept, the measurements were set-up as in Figure 2, where Figure 2a shows the simulated boundary conditions (X-axis: applied electric field, Y-axis: applied magnetic field), Figure 2b represents the necessary experimental components and accessories.

## 3. Absorption and Pressure Sensor Results and Discussion

### 3.1. E-Field, H-Field and Surface Current Analysis

Before we start the analysis, some prominent research papers must be consulted to explore the unit cell behavior. At the visible frequency range, a collaborative research group from National University of Singapore (NUS) have proposed an ultrathin flat bilayer metasurface design to work over the broadband range [38]. An interesting feature of surface wave application is control and manipulation of electromagnetic wave propagation that can achieve cloaking features [39]. Herein, we have analyzed the design according to basic field distribution, specifically the voltage and current distribution.

The EM field distribution at maximum absorption rating (at resonance frequency), which is 99.6%, has to be explained regarding both physics and mathematics. To do so, we will consider the well-known Helmholtz equation or the vector wave equations for both the E-field and the H-field. We know that:

(6) $$\nabla^{2}E_{m}-\gamma^{2}E_{m}=0$$

where, *E_m_* = E-field propagation or distribution component

*γ* = propagation constant,

where, $\left|\gamma^{2}\right|=\omega \mu \sqrt{\sigma^{2}+\omega^{2}\varepsilon^{2}}$.

**Figure 3:**
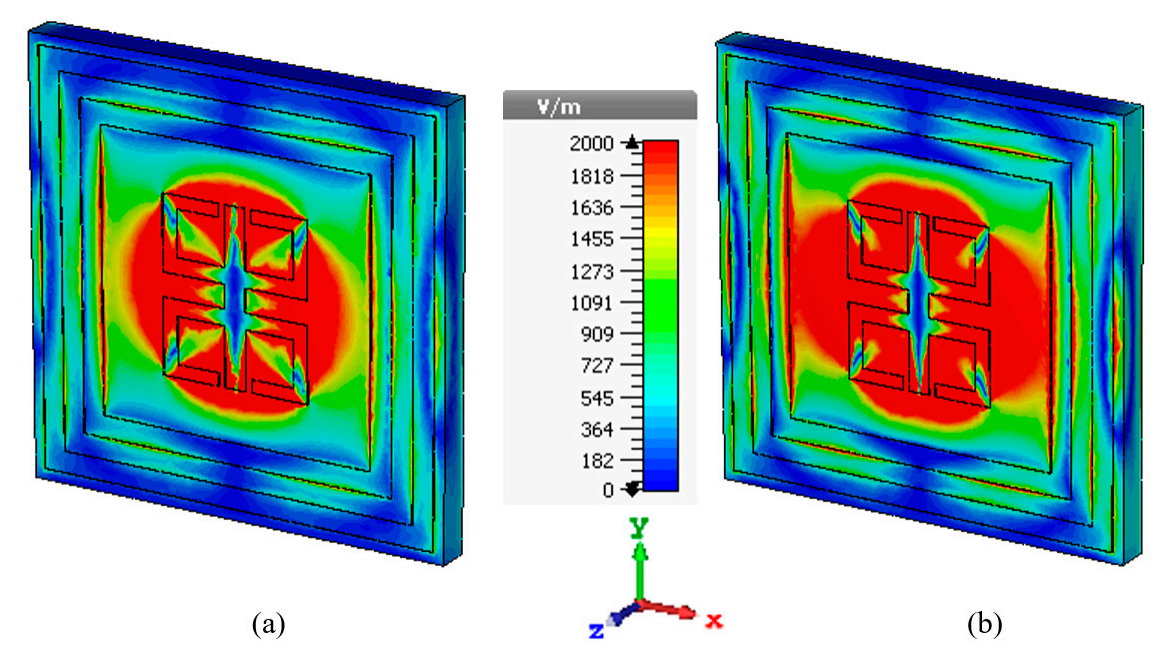
E-field distribution at the resonance frequency (**a**) 13.78 GHz, (**b**) 15.3 GHz of proposed MM absorber.

According to the Helmholtz equation (assuming standard conditions), both the E-field and the H-field propagation are dependent on the propagation constant γ of the medium. Also, *γ* is dependent on permittivity (*ε*), permeability (*µ*), frequency (*ω*), and conductivity (*σ*) as we can see in the Equation (6). The proposed metamaterial absorber substrate FR-4 has *ε* = 4.6 and *µ* = 1.0. Combing all these parameters, Equation (6) reduces to:

(7) $$\left[\frac{d^{2}}{dz^{2}}-\gamma^{2}\right]E_{xm}(z)=0$$

which is a linear homogeneous differential equation. Taking a trivial solution, it gives two components of this E-field, so, at resonance frequency, say at 13.78 GHz, assuming the wave propagation along the +*z* direction it gives an optimum distribution around the inner mirror reflexed C-shape resonator. Not only that, some E-field components are also present at the vertical edge of the two-ring resonator at the outer side of the unit cell absorber. Highly dense electric fields couples mutually with each other to form a strong dipole moment. This moment is equally shared by the upper and lower split sections at the resonance frequency as shown in Figure 3a.

As the unit cell has another resonance at 15.3 GHz with the absorption of 99.14%, the same momentum sharing occurs here again as shown in Figure 3b and rest of the frequencies, this E-field distribution starts to scatter along the rings.

**Figure 4:**
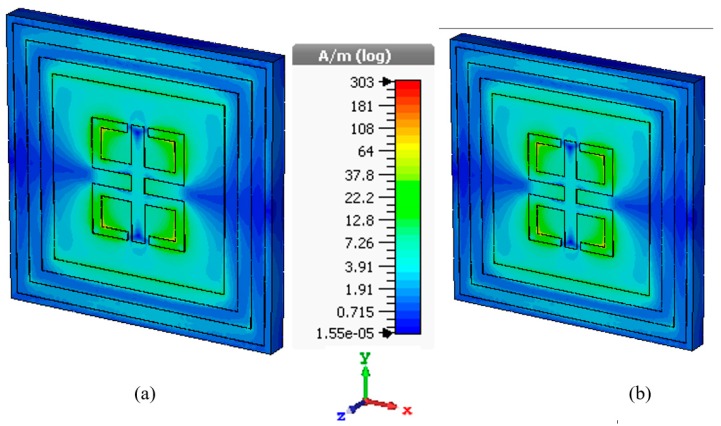
H-field distribution at the resonance frequency (**a**) 13.78 GHz, (**b**) 15.3 GHz of proposed MM absorber.

Figure 4a,b shows the magnetic field distribution at the same frequency as mentioned above for the E-field distribution. For the maximum absorption, the unit cell has a few interesting properties like observed left-handed metamaterial (LHM) characteristics with values listed in Table 2. LHM has double-negative property means a metamaterial made of a repeated lattice of conducting, non-magnetic elements that exhibits effective permittivity and permeability both are simultaneously negative over a band of frequencies [40]. This type of medium has been termed ‘left-handed’ as the electric field, magnetic field and propagation vector (or wave number) are related by a left-hand rule.

As for the EM wave shown in Equation (1), a similar expression is valid for magnetic field propagation or distribution, like:

(8) $$\nabla^{2}H_{m}-\gamma^{2}H_{m}=0$$

where *H_m_* = H-field propagation or distribution component.

The similar trivial solution gives two components of the H-field where both of them couple to each other in the outer two rings as well as in the mirror reflexed C-shape portion in the inner side. It is evident from Figure 4 that, a split and vertical section of the inner resonator gives a significant response at the resonance frequency. Also, the magnetic dipole moment created at that instant shows a highlighted portion which approximately ranges from 10 to 24 A/m in a logarithmic scale. Similar to E-field distribution, the H-field propagation constant is dependent on *ε*, *µ*, and *σ* for which field distribution become stable at the resonance frequency with LHM properties.

**Figure 5:**
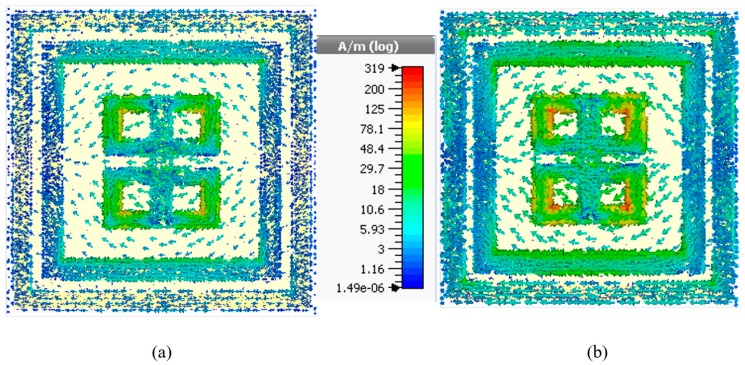
Surface current distribution of proposed MM absorber (**a**) 13.78 GHz, (**b**) 15.3 GHz.

Surface current distributions are shown in Figure 5a,b, where two distinct distributions with high density are observed at the resonance frequency. At 13.78 GHz and 15.3 GHz, the outer and inner resonators have a significant amount of surface current roaming around the edges. An important point to remember is that, the microstrip pattern uses Cu (thickness of 0.035 mm) on top of a FR-4 substrate, so a traveling wave may face a situation where at low frequency the wave has a good conductor medium but with increasing frequency the dielectric property of the same medium decreases. In order to explain such surface, current distribution at these specific frequencies some basic equations like Ampere’s law for EM field should be recapped:

(9) $$\nabla \times \vec{H}=\vec{J}+\frac{\partial \vec{D}}{\partial t}$$

where, $\vec{J}$ = conduction current density

$\frac{\partial \vec{D}}{\partial t}$ = displacement current density = $\vec{J_{d}}$.

The currents $\vec{J}$ and $\vec{J_{d}}$ form the loss tangent tanθ, $\tan\theta =\frac{\sigma }{\omega \varepsilon }$. On the other hand, patching of unit completed with Cu (which is a good conductor) also plays a role for this surface current. As we know, when an E-field or H-field wave travels in a conducting medium, its amplitude is attenuated by the factor $e^{-\alpha z}$ where, α is the attenuation constant. The distance the wave travels till attenuation is called skin depth or penetration depth, $\delta =\frac{1}{\alpha }$, so as the MM absorber reaches the resonance frequency, this loss angle moves to its maximum value while curving the traveling wave at the edge of each patch portion. Moreover, Equation (9) indicates that the maximum loss tangent will enhance the right-hand side of the equation to show the dominant value of the magnetic field. Additionally, in the GHz range frequency, copper shows a skin depth of approximately 6.6 × 10^−4^ mm and the skin depth decreases as the frequency increases [41]. That is why both mirror reflexed C-shaped inner resonators have high values of surface current.

### 3.2. Quasi-TEM Polarization Independence Regarding Maxwell Equations

**Figure 6:**
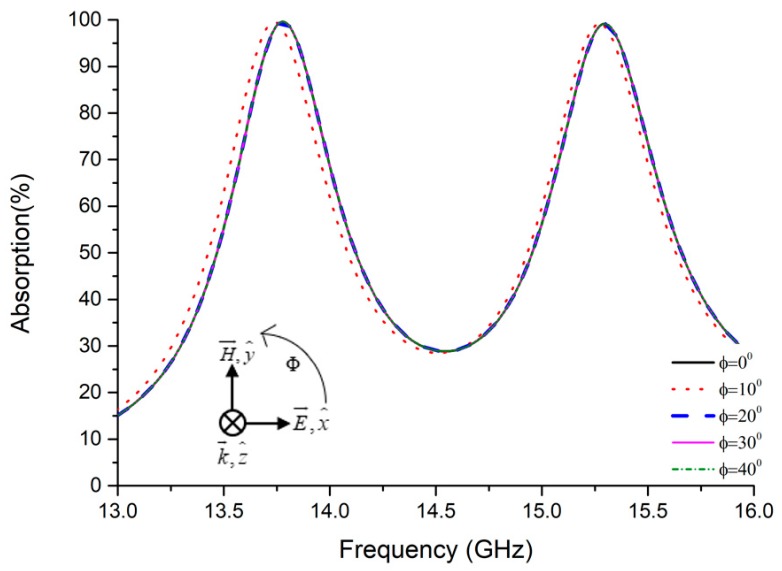
Absorption of quasi-TEM polarization independence of the proposed absorber.

For microstrip lines or patches, the TEM properties have some slightly propagation differences. As we know, TEM has transverse characteristics of EM wave, but as for a general microstrip, the EM wave propagates through the air above the top pattern and the dielectric substrate. As a result, in two different media having different resistivity the wave propagates at different speeds in both the regions. This is referred to as quasi-TEM mode. Conventionally, the EM wave propagation as depicted in Equation (7) can be represented in regarding voltage and current like:

(10) $$\begin{array}{l} \frac{d^{2}V}{dz^{2}}-\gamma^{2}=0\\ \frac{d^{2}I}{dz^{2}}-\gamma^{2}=0 \end{array}\}$$

where, *γ* is dependent on ε, µ, ω, and σ as shown in Equation (6). On the contrary, the propagation constant *γ* = $\sqrt{Z^{\prime }Y^{\prime }}$ as the wave propagation is characterized by a traveling wave. Here, $Z^{\prime }$ and $Y^{\prime }$ are the transmission line normalized impedance and admittance of the line per unit length, respectively. Now, if we observe the surface current distribution in Figure 5, the inner and the outer resonator have contours in adjacent fields. That is why Equations (6) and (10) have a slight variation in expression which can be approximated as [42]

(11a) $$\frac{dE_{x}}{dz}=-Z^{\prime }H_{y}=-j\omega \mu H_{y}$$

(11b) $$\frac{dH_{y}}{dz}=-Y^{\prime }E_{x}=-j\omega \varepsilon E_{x}$$

so, the value of impedance and admittance can be further modified using the circuit diagram shown in Figure 1a and if the transmission line is not so long, propagation of a wave can be treated as quasi-TEM. In Figure 6 the absorption of this quasi-TEM propagation of proposed MM absorber is shown.

### 3.3. Absorption Based on Simulation

**Figure 7:**
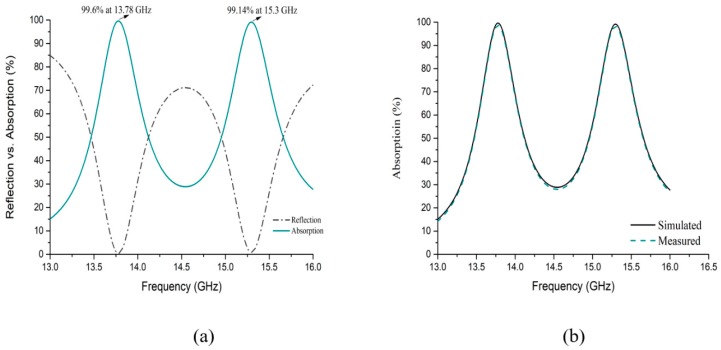
(**a**) reflection vs. absorption performance, (**b**) simulated and measured absorption of proposed MM absorber.

In this proposed metamaterial absorber, unit cell design, analysis, and simulation were performed on CST Microwave Studio using the FIT method. Boundary conditions were applied periodically on the waveguide port. All other conditions like substrate thickness, patching pattern, outer and inner resonator spacing have been analyzed based on the design methodology. In Figure 7a, simulated absorption as well as a reflection of EM energy concerning frequency are shown. As from Equation (3), it is evident that absorption depends on the admittance formed by the patch from the equivalent circuit considered for the prototype.

Peak absorption (Figure 7a) is rated at 99.6% and 99.14% with a respective frequency of 13.78 GHz and 15.3 GHz in the presence of LHM properties. Theoretically, a perfect absorber should have zero reflection (R = 0) to get an ideal absorption of 100% at the resonance frequency. In the experimental measurement, simulated absorption and measured absorption have proximity as depicted in Figure 7b. It is evident that measured data have roughly 5~10% variation than the simulated one at the resonance frequency.

As the MM absorber simulation shows two resonance frequency (13.78 GHz and 15.3 GHz), energies at that time dissipated by the substrate and patch model on it, so the energy consumption may result in heating on the absorber. For the time being this factor was not considered in the proposed model, but a non-linear recursive feedback optical-thermodynamic numerical analysis in [43] showed that optical absorption induced temperature affects metamaterial absorber performance. However, the inner and outer resonator model have a mutually coupled E-field and H-field region at the splits and between ring resonators. Another significant point is that the proposed absorber shows quasi-TEM properties with LHM characteristics which are not available in usual material absorbers.

Figure 1c depicts that the unit cell has a layer of the dielectric substrate placed between quasi-TEM systems. We know a relationship from Rothwell and his research group [44] that was used to study Nicolson-Ross-Weir (NRW) method, which is:

(12) $$S_{21}=js_{0}S_{11}M$$

where, $s_{0}$ = ±1 and $M=\sqrt{\frac{1-{\left|S_{11}\right|}^{2}}{{\left|S_{11}\right|}^{2}}}$.

**Figure 8:**
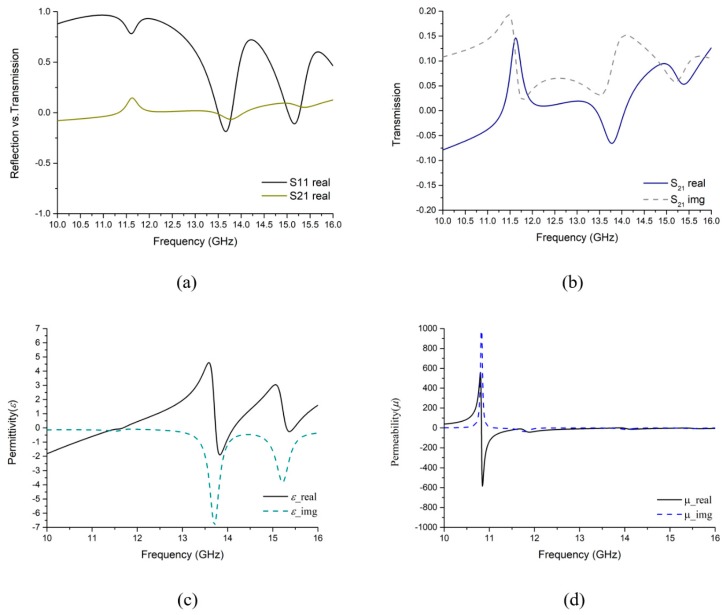
Reflection and Transmission properties in simulated design (**a**) Reflection vs. Transmission, (**b**) Transmission characteristics, (**c**) Permittivity (*ε*), (**d**) Permeability (*µ*) with Frequency spectrum.

**Figure 9:**
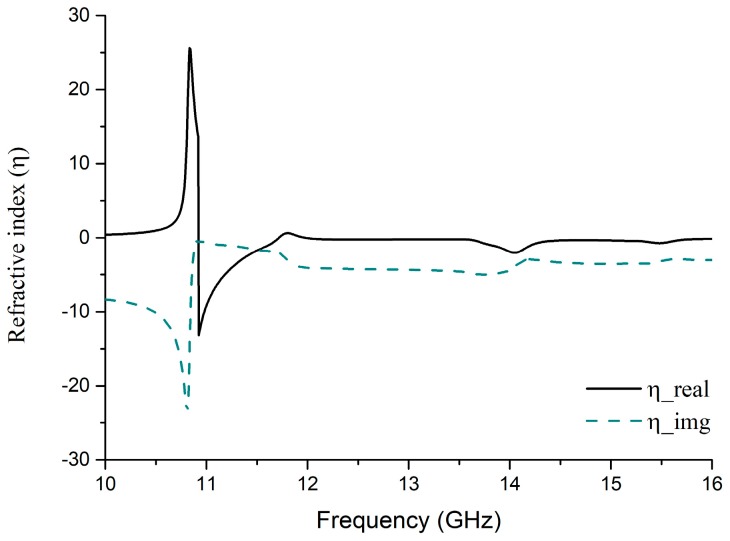
Refractive Index of designed MM absorber in a simulated environment using DRI method.

So, if there is any change in polarity of $s_{0}$ then the real value of $S_{11}$ remains unchanged. Producing four distinct symmetric polarization angles φ which vary from −π to +π having no identical value. As a result, Equation (12) and Figure 8a,b illustrate that at the resonance frequency, especially at 13.78 GHz, both transmission ($S_{21}$) and reflection ($S_{11}$) real parts, have negative values of *ε* (−1.48) and *µ* (−0.36) (in Figure 8c,d) that signify DNG metamaterial properties. Similarly, at 15.3 GHz, positive values of transmission and reflection gives negative permittivity and permeability, but at this point, the permittivity becomes very close to positive. On the other hand, based on the *S*-parameter retrieval from the simulated environment of the proposed MM absorber, the refractive index (ν) performance is being shown in Figure 9.

The traditional Direct Refractive Index (DRI) method [45] was used for calculating the parameter which is:

(13) $$\nu \approx \frac{c}{j\pi fh^{\prime }}\sqrt{\frac{\left[{\left(S_{21}-1\right)}^{2}-S_{11}{}^{2}\right]}{\left[{\left(S_{21}+1\right)}^{2}-S_{11}{}^{2}\right]}}$$

where, *f* represents the frequency and *h*′ is the thickness of the substrate. At the resonance frequency of both 13.78 GHz and 15.3 GHz, it clearly presents negative values of −0.887 and −0.460, respectively.

A comparative study based on simulation of the proposed MM absorber, is given in Table 3 where the FR-4 material shows significantly good performance regarding absorption capacity compared to Rogers RT-5880 substrate. Moreover, a dual-band resonance with wide bandwidth (13.67–13.88 GHz, 15.19–15.4 GHz approx.) gives a flexibility option in FR-4 compared to RT-5880 where it shows a narrowband absorption. Additionally, the proposed absorber shows LHM properties which can be apply in sensing applications and the cost of this material is low compared to Rogers RT-5880 or other available materials.

### 3.4. Pressure Sensor on Simulation Environment

**Figure 10:**
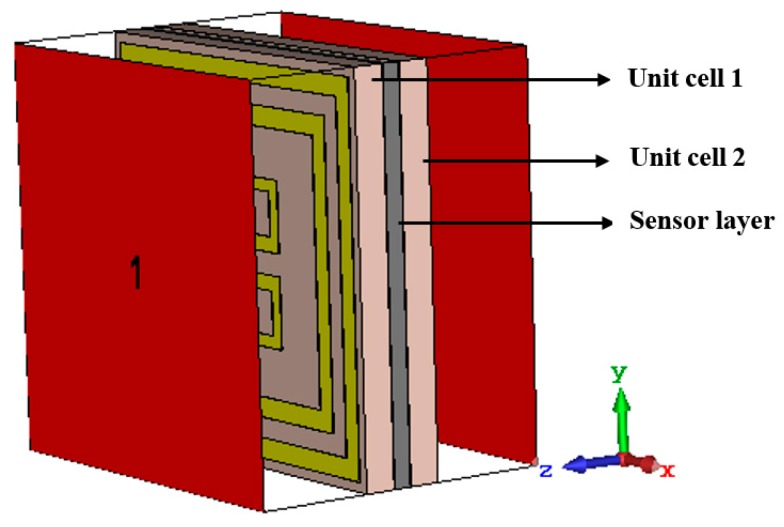
Simulation setup for a pressure sensor using metamaterial absorber unit cell.

Extensive studies have been done in metamaterial absorbers in the last few years. For instance, single nanoparticle and nanorod devices for biosensing application, radar cross section (RCS) control for antenna design based on metamaterials, optical nanocircuits inspired by metamaterials, surface wave cloaking from gradient index materials to use nanocomposites in EM wave control, ultrathin multicolor metasurfaces for high resolution spatial light modulators [46,47,48,49,50]. Despite such contributions, this unit cell has a new design with inner and outer resonators with polarization independent characteristics. This feature was studied applying quasi-TEM mode wave propagation which makes this design more versatile in X and Ku band applications. Also, dual band resonance with high absorption rating and sensing characteristics in a simulation environment is a notable point for this design. Moreover, the second resonator inside the lattice reduces the form factor which has significant value for absorber design. On the other hand, the dimension of the unit cell were optimized for X and Ku band pressure sensing applications. In Figure 10, a simulation arrangement where two unit cells sandwiched with a sensor layer is depicted. Each unit cell has dielectric substrate FR-4, a metamaterial absorber (MMA) ring with a mirror reflexed C-shape and a copper ground plane. Rogers RT 5880 (ε = 2.2, μ = 1.0), RT 5870 (ε = 2.33, μ = 1.0) and RT 6202 (ε = 2.94, μ = 1.0) these three high frequency circuit materials were studied in our simulations as sensor layers. RT 5880/5870 are filled with polytetrafluroethene (glass or ceramic) composite specifically laminated for high reliability, aerospace and defense systems, millimeter wave applications, space satellite transceivers, etc. Waveguide ports 1 and 2 are used for field excitation.

**Figure 11:**
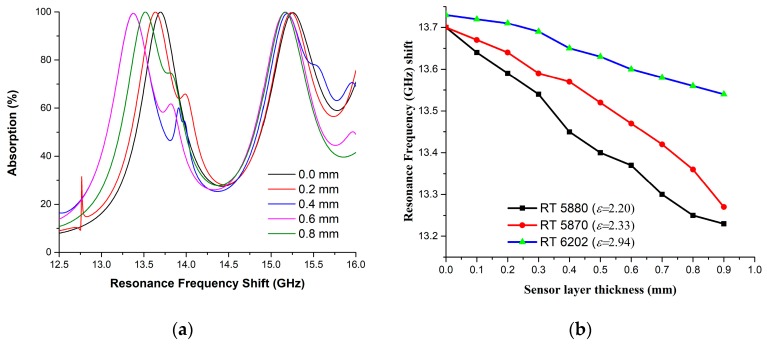
Unit cell absorber for pressure sensitivity in simulation (**a**) Absorption (%) amount and Resonance frequency shift w.r. to sensor layer thickness (**b**) Sensor layer relationship with Resonance frequency.

To evaluate the unit cell for pressure sensitivity through simulation, the sensor layer thickness was considered to start from 0.0 mm and gradually increased by 0.1 mm/observation up to 0.9 mm. Figure 11 depicts that the sensor layer thickness change is inversely proportional to the resonance frequency shift. It is notable to mention that, for simplicity of shifting sequence representation data plotting was performed with 0.2 mm/observation and data extraction from the simulation was based on 0.1 mm/observation. Hence, this unit cell shows an average shifting of resonance frequency approximately 52 MHz/observation for Rogers RT 5880 sensor layer material. Similar steps have been followed for RT 5870 and RT 6202 as sensing layer materials and the results are plotted in Figure 11b. Additionally, a comparison with other sensing applications of MM absorbers is shown in Table 4. Despite using the same widely used substrate (FR-4) this unit cell absorber has a reduced size with significant absorption and a notable sensitivity to thickness changes. Comparing this unit cell with others, it would be useful for sensing applications for satellite communication and radar systems.

## 4. Conclusions

A metamaterial absorber (MMA) based on a Ring with mirror reflexed C-shape has been presented for sensing applications in the X and K_u_ band. Design and fabrication of the absorber have been compared through simulations and measurements, to validate the performance and analytical data. Though there are two resonators the balanced mutual surface current distribution had a good impact on performance, creating a dual band resonance. A parametric analysis has been performed between FR-4 and Rogers RT-5880 based on this prototype to identify the applications of the proposed band. Unit cell fabrication shows the design compactness in size as well as physical orientation for better understanding. Having mentioned all these positive facts about this study a few limitations persist here also. Measuring the absorption rating in the measurement and simulation environment based on unit cell performance though an array design would be more accurate regarding practical applications. The experimental procedure is more accurate than simulation for pressure sensing characteristics evaluation. Further studies could be conducted concerning error minimization, especially in the inverse relationship of thickness and resonance frequency shifting. Flexible substrates would be another option to design absorbers with sensing application in the future to make the application field more versatile, nevertheless, a high absorption rate signifies the potential of this absorber for sensing in satellite applications, microwave shielding, and imaging systems.

## Figures and Tables

**Table 1:** Dimension of Ring C-shaped quasi-TEM absorber.

Parameter	*l*_1_	*l*_2_	*l*_3_	*h*_1_	*h*_2_	*h*_3_	*w*_1_	*w*_2_	*w*_3_	*w*_4_	*g*_1_	*g*_2_	*g*_3_
**Size (mm)**	19	15	2.70	19	15	3.50	1	1	1	0.70	0.30	1.10	1
	Substrate length & width, *S*												
	20												

**Table 2:** LHM characteristics values of proposed MM absorber at resonance.

Resonance Frequency (GHz)	Permittivity, *ε*	Permeability, *µ*	Reflection Co-Efficient, *η*
13.78	−1.484	−0.362	−0.887
15.3	−0.048	−1.054	−0.460

**Table 3:** Parametric characteristics study of proposed MM absorber at the resonance frequency.

Polarization Angle (*φ*)	Resonance Frequency (GHz)	Max Absorption (%)	*ε*	*µ*	ν	EM Mode	Substrate	Dielectric Constant (*ε_r_*)
0	13.78	99.6	−1.484	−0.361	−0.887	TEM	FR-4 (lossy)	4.6
	15.3	99.14	0.002	−1.054	−0.460			
10	13.74	99.52	−1.601	−0.343	−0.885	TEM	FR-4 (lossy)	4.6
	15.27	99.1	0.073	−1.114	−0.4466			
20	13.78	99.6	−1.484	−0.361	−0.887	TEM	FR-4 (lossy)	4.6
	15.3	99.14	0.002	−1.054	−0.460			
30	13.78	99.6	−1.484	−0.361	−0.887	TEM	FR-4 (lossy)	4.6
	15.3	99.14	0.002	−1.054	−0.460			
40	13.78	99.6	−1.484	−0.361	−0.887	TEM	FR-4 (lossy)	4.6
	15.3	99.14	0.002	−1.054	−0.460			
50	13.78	99.6	−1.484	−0.361	−0.887	TEM	FR-4 (lossy)	4.6
	15.3	99.14	0.002	−1.054	−0.460			
60	13.78	99.6	−1.484	−0.361	−0.887	TEM	FR-4 (lossy)	4.6
	15.3	99.14	0.002	−1.054	−0.460			
0	12.95	91.39 (very narrow band)	−1.327	−4.437	−1.515	TEM	RT-5880 (lossy)	2.2
10	12.95	91.39	−1.327	−4.437	−1.515	TEM	RT-5880	2.2
20	12.95	91.39	−1.327	−4.437	−1.515	TEM	RT-5880	2.2
30	12.95	91.39	−1.327	−4.437	−1.515	TEM	RT-5880	2.2
40	12.95	91.39	−1.327	−4.437	−1.515	TEM	RT-5880	2.2
50	12.95	91.39	−1.327	−4.437	−1.515	TEM	RT-5880	2.2
60	12.95	91.39	−1.327	−4.437	−1.515	TEM	RT-5880	2.2

**Table 4:** Comparison of the MM absorber in other sensing applications.

Ref. #	Size (mm)	Substrate Material	Operating Frequency [GHz]	Application	Remarks
[25]	24 × 30	FR-4	3–6	Temperature sensor	Absorption rate can be tuned
[26]	20 × 20	FR-4	2–17	No sensing application	Six-band metamaterial absorber
[27]	36 × 36	FR-4	2–6	Permittivity sensor	Perfect absorption
[29]	28 × 35	FR-4	3–5	Micro-fluid sensor	Metamaterial based sensing no absorption
[30]	40 × 20	F4B	Less than 1	Permittivity sensor	Metamaterial based sensing no absorption
[28]	24 × 24	FR-4	3–5	Refractive index sensor	Ring resonant absorber
[31]	60 × 40	RT 5880	7–8	Chemical sensor	Single band with same liquid loading system
Proposed	20 × 20	FR-4	X and Ku Band	Pressure sensor	Dual-band Perfect Absorber and sensing simultaneously
